# Ethylmalonyl-CoA pathway involved in polyhydroxyvalerate synthesis in *Candidatus Contendobacter*

**DOI:** 10.1186/s13568-022-01380-3

**Published:** 2022-03-25

**Authors:** Chen Zhao, Chunchun Zhang, Zhiqiang Shen, Yanping Yang, Zhigang Qiu, Chenyu Li, Bin Xue, Xi Zhang, Xiaobo Yang, Shang Wang, Jingfeng Wang

**Affiliations:** 1 Department of Hygienic Toxicology and Environmental Hygiene, Tianjin Institute of Environmental and Operational Medicine, Tianjin, China; 2 School of Environmental Science Engineering, Tiangong University, Tianjin, China

**Keywords:** Glycogen-accumulating organism, Propionyl-CoA, PHV, EMC pathway

## Abstract

Here a stable glycogen accumulating organisms (GAOs) system was operated by anaerobic–aerobic mode in the sequencing batch reactor. We focused on the metabolic mechanisms of PHAs storage from GAOs. Our system showed the classic characteristic of glycogen accumulating metabolism (GAM). Glycogen consumption was followed by acetic acid uptake to synthesize poly-β-hydroxyalkanoates (PHAs) during the anaerobic period, and glycogen was synthesized by PHAs degradation in the aerobic stage. Microbial community structure indicated that *Candidatus Contendobacter* was the most prevalent GAOs. We found that the ethylmalonyl-CoA (EMC) pathway was the crucial pathway supplying the core substance propionyl-CoA for poly-β-hydroxyvalerate (PHV) synthesis in *Candidatus Contendobacter*. All genes in EMC pathway were mainly located in *Candidatus Contendobacter* by gene source analysis. The key genes expression of EMC pathway increased with *Candidatus Contendobacter* enrichment, further validating that propionyl-CoA was synthesized by *Candidatus Contendobacter* predominantly via EMC pathway. Our work revealed the novel mechanisms underlying PHV synthesis through EMC pathway and further improved the intercellular storage metabolism of GAOs.

## Key points

- We observed GAM characteristic in the GAOs enrichment system.
- Metagenome-based analysis revealed that *Candidatus Contendobacter* was the dominant GAOs.
- The EMC pathway was a novel propionyl-CoA synthesis pathway for PHV in *Candidatus Contendobacter*.

## Introduction

Enhanced Biological Phosphorus Removal (EBPR) is a widely used process for achieving phosphorus removal from wastewater. Phosphorus-accumulating organisms (PAOs) play a major role for EBPR. Under anaerobic conditions, PAOs absorb volatile fatty acids (VFAs) and simultaneously synthesize the intercellular storage substance-PHAs, the intracellular polyphosphate (poly-P) degradation can also provide energy in the phase. In the subsequent aerobic phase, PAOs consume PHAs for growth, glycogen replenishment, and phosphorus absorption (Carvalho et al. 2010; Vargas et al. 2013; Zhou et al. 2008). However, the change of operating parameter, such as carbon-phosphorus ratio, substrate type, temperature and redox potential changes, can lead to inefficiencies and even the deterioration of EBPR system (Nittami et al. 2017; Tayà et al. 2013; Welles et al. 2016a; Zhang et al. 2008). The problems have often been attributed to glycogen accumulating organisms (GAOs), although this is yet to be convincingly shown in full-scale systems where the presence of GAOs does not always coincide with poor performance (Lopez-Vazquez et al. 2009; Weissbrodt et al. 2013; Winkler et al. 2011). GAOs raise growing concern about the adverse impacts of EBPR system. Similar to PAOs, GAOs consume their intracellular glycogen as the energy source for VFAs absorption and PHAs synthesis. In the following aerobic phage, GAOs consume PHAs to synthesize glycogen for the growth (Zhang et al. 2008). However, some studies have reported that GAOs exit in full-scale EBPR plants and not affect the phosphorus removal efficiency (Lanham et al. 2013; Nielsen et al. 2019). Therefore, the biological characteristics of GAOs need further explore.

The intracellular storage substances play a key role in regulating the metabolism of microbiology. PHAs including poly-β-hydroxybutyrate (PHB), poly-β-hydroxyvalerate (PHV) and poly-β-hydroxy-2-methylvalerate (PH2MV) are the important intracellular carbon and energy sources for GAOs as well as PAOs. In comparison with PAOs, GAOs are more active in terms of PHA utilization and produce more diverse PHAs (PHB and PHV) with the same organic substrate (Bengtsson 2009; Zhang et al. 2019). In GAOs, PHAs synthesis is involved in the following pathway: Acetic acid is consumed to form acetyl-CoA, and propionyl-CoA is produced from pyruvate (an intermediate in glycolysis) through the succinate-propionate pathway. Finally, one molecule of acetyl-CoA and propionyl-CoA is polymerized to PHV under the action of polymerase, while two molecules of propionyl-CoA form 3-hydroxy-2-methylvalerate and then are polymerized to PH2MV. Many studies validated that GAOs produced propionyl-CoA mainly through the succinate-propionate pathway and then combines with acetyl-CoA to form PHV. However, based on the difference of bacterial species, propionyl-CoA can be produced via different routes, not just via the succinate–propionate pathway (Guedes da Silva et al. 2020; McIlroy et al. 2014; Schneider et al. 2012). Hence, the metabolisms of the intercellular storage substances in GAOs still remain unclear.

There may be significant differences in glycogen degradation, intercellular storage and VFA metabolism among different kinds of GAOs (McIlroy et al. 2014; Oehmen et al. 2006). For example, there were considerable differences between *Candidatus Contendobacter denitrificans* and *Candidatus Competibacter odensis* for the Embden-Meyerhof-Parnas and Entner-Doudoroff glycolytic pathways, *Candida contedobacter* cannot carry out glycolysis through Entner-Doudoroff pathway or denitrification (McIlroy et al. 2014). Many studies have reported that the denitrifying GAOs become one of the crucial functional microorganisms in full-scale EBPR plants with stable performance (Ji et al. 2017; Yuan et al. 2020). GAOs have also been used for the simultaneous removal of pollutants such as nitrogen and phosphorus under anaerobic–aerobic conditions (He et al. 2020). In addition, some studies investigated the feasibility of simultaneous partial nitrification, denitrification, and phosphorus removal in a single-stage anaerobic/microaerobic sequencing batch reactor (Yuan et al. 2020), suggesting that denitrifying GAOs and denitrifying PAOs played a major role in nitrogen and phosphorus removal, respectively. Therefore, the intercellular storage substances play a vital role in the metabolism of GAOs, such novel applications need further uncover mechanisms on the intercellular storage and the different functions.

In this study, we developed a stable GAO enrichment model-based anaerobic–aerobic sequencing batch reactor (SBR) system in which acetic acid was used as the sole carbon source and decanting after the anaerobic period. Metagenome and metatranscriptome were used to study the dynamic changes of microbial community. We focused on analyzing and identifying the various pathways and the key genes with the metabolisms of intercellular storage substances. Further, we investigated the key genes source to assess the core pathway of intercellular storage metabolism in GAOs. Our results would likely increase our ability to optimize, manipulate and extend the bioprocess of GAOs enrichment system.

## Materials and methods

### Reactor design and operation process

**Fig. 1:**
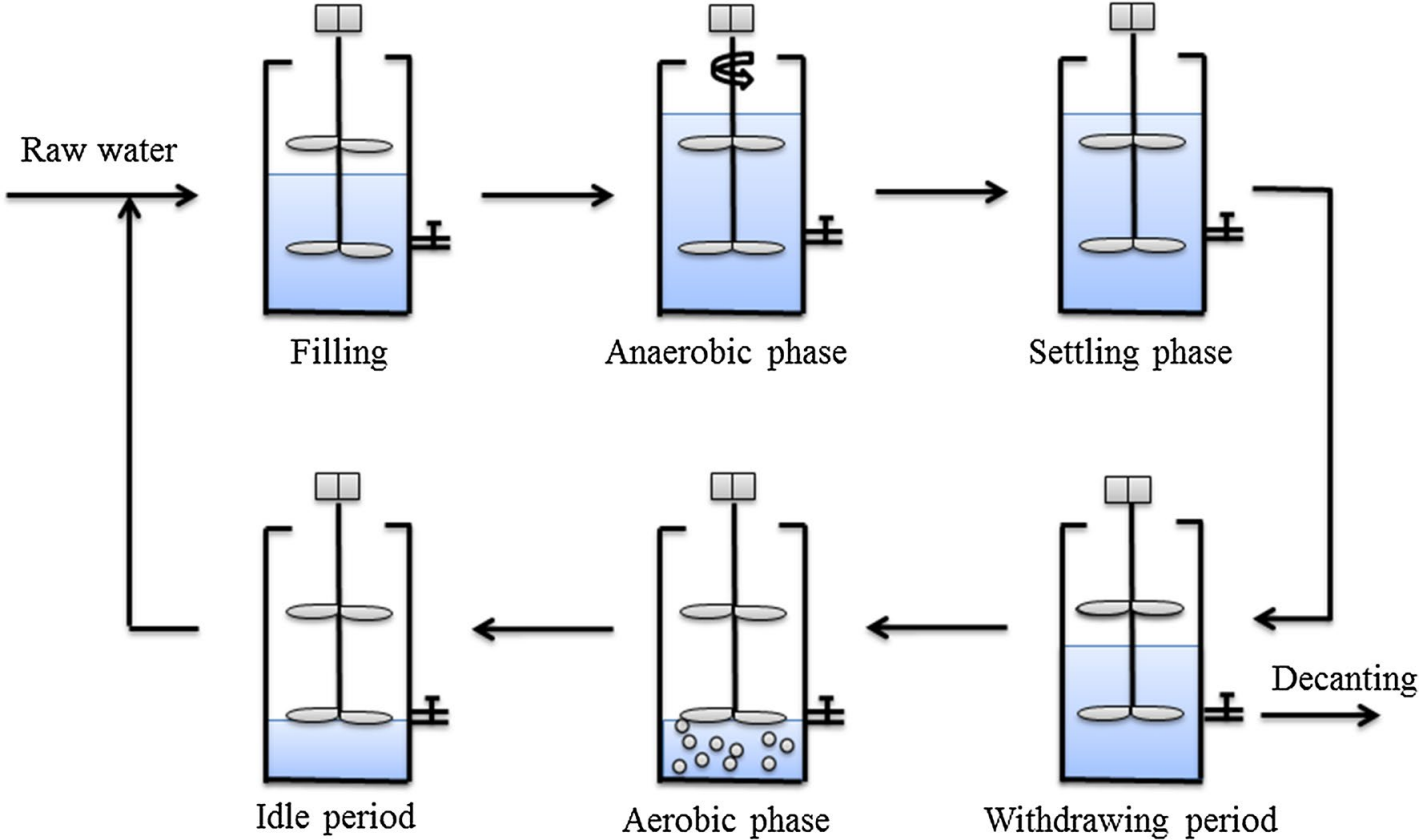
Schematic diagrams of SBR operation

SBR system was operated under anaerobic–aerobic conditions. The inoculated sludge came from the secondary sedimentation unit of Sewage Treatment Plant, Tianjin, China. The volume exchange rate was approximately about 70% after the anaerobic stage, and the cycle time was 6 h. Each cycle consisted of six stage including filling period (2 min), anaerobic phase (90 min), settling phase (15 min), withdrawing period (6 min), aerobic phase (240 min), idle period (9 min), the reactor operation flow is shown in a single cycle (Fig. 1). The SBR system was controlled using a programmable logic controller, input water was controlled by a liquid level meter and a submersible pump. Further, anaerobic mixing was achieved using an electric agitator, and aeration was controlled with a rotameter.

### Culture media

In the system, we used synthetic wastewater, which included 400–440 mg HAc/L as the source of carbon, 40 mg/L NH_4_^+^-N (provided by NH_4_Cl) as the source of nitrogen, 5 mg/L PO_4_^3^-P (provided by KH_2_PO_4_) as the source of phosphorous. Other nutrient salts included 50 mg/L MgSO_4_·7H_2_O, 20 mg/L KCl, 20 mg/L CaCl_2_, 0.1 mg/L FeSO_4_·7H_2_O, 0.1 mg/L CuSO_4_·5H_2_O, 0.1 mg/L MnSO_4_. According to the properties of the actual wastewater, pH was not controlled in this study.

### Analytical methods of reactor performance

Acetic acid levels were assessed with high performance liquid chromatography using an ODS-2 HypersilTM column (Thermo Fisher Scientific, Waltham, MA, USA) and ultraviolet light detector (Qing 2017). PHAs (PHB and PHV) levels were assessed with gas chromatography (GC) (Oehmen et al. 2005). Weighed freeze-dried biomass and PHB/PHV standards were placed into the glass tubes and heated at 105 ℃ for 6 h after being mixed with 2 mL methanol acidified with 3% H_2_SO_4_ and 2 mL chloroform. Benzoic acid was used as internal standard. After cooling, 1 mL Milli-Q water was added to the samples, followed by thorough mixing and incubation at − 20 ℃ for overnight to achieve phase separation. After centrifugation for 5 min at 6000 rpm, PHA levels in the chloroform phases (bottom layer) were measured. Glycogen levels were examined according to the previous method (Wang et al. 2006). Intracellular glycogen was extracted using NaOH and C_2_H_5_OH after extracellular polymer removal at 70 ℃, and then the anthrone method was used for glucose concentration measurement. Phosphate levels were measured using ammonium molybdate spectrophotometry. Mixed liquor suspended solids were assessed using gravimetric method.

### Metagenomic sequencing

Samples were collected at the end of the anaerobic period on days 1, 20, 39, 52 and 75 of sludge inoculation at regular intervals for three consecutive cycles. 5 mL mud-water mixed samples were collected during each cycle period. DNA was extracted and purified. A total of 1 μg DNA per sample was used to generate sequencing libraries whose inserts size was about 350 bp. Library construction and sequencing were completed on the Illumina HiSeq platform (Novogene Bioinformatics Technology Co, Ltd., Beijing, China). The raw data obtained was processed using Readfq (V8, https://github.com/cjfields/readfq) to acquire the clean data for subsequent analysis. Gene abundance evaluation and species annotation were eventually performed. The method details were described in the previous studies (Ai et al. 2019; McIlroy et al. 2014). The metagenomic sequencing data have been submitted to NCBI Sequence Read Archive database under accession numbers PRJNA792335.

### Metatranscriptome sequencing

Sampling was performed as described above. Total RNA was extracted using TRIzol (Invitrogen, Waltham, MA, USA) and quality checked using 1% agarose gel electrophoresis and a Qubit 2.0 fluorometer (Invitrogen). RNA integrity and quantity were finally measured using RNA Nano 6000 Assay Kit of the Bioanalyzer 2100 system. RNA library for metatranscriptome-seq was prepared as rRNA depletion and stranded method. RNA was fragmented into 250 ~ 300 bp fragments and reverse transcribed into cDNA. Remaining overhangs of double-strand cDNA were converted into blunt ends via exonuclease/polymerase activities. After adenylation of 3’ ends of DNA fragments, sequencing adaptors were ligated to the cDNA. In order to select cDNA fragments of preferentially 250 ~ 300 bp in length, the library fragments were purified with AMPure XP system. Amplification of cDNA was performed using PCR. Raw data generation, data filter and taxonomy annotation were the same as metagenomic sequencing. The metatranscriptome sequencing data have been submitted to NCBI Sequence Read Archive database under accession numbers PRJNA791813.

### Data analysis for the microbial community and functional genes source

To annotate microbial community in the system, the sequences of bacteria were searched against NR database (Version: 2018-01-02, https://www.ncbi.nlm.nih.gov/) of NCBI for microbial community identification using DIAMOND (Buchfink et al. 2015) blastp, with a cut-off e-value of 1e−5. As each sequence might aligned to multiple results, the result with the e-value ≤ the smallest e-value *10 was chosen to take the LCA (lowest common ancestor) algorithm to make sure the species annotation of sequences (Hanson et al. 2016).

For analyzing functional genes source, DIAMOND software with the same cut-off value was used to blast Unigenes to the KEGG database (Version 2018-01-01, http://www.kegg.jp/kegg/). For each sequence’s blast result, the best Blast Hit was used for subsequent analysis. If the functional genes and taxonomy information were located in the same Unigenes, it was considered that the functional genes were contributed by this taxonomy. Subsequently, the relative abundance of different functional hierarchy and the gene number table of each sample in each taxonomy hierarchy were obtained. Then the top five hosts in terms of abundance were selected for the next step of analysis by ranking the host abundance of the annotated functional genes.

## Results

### Establishment of GAOs reactor and performance indexes

**Fig. 2:**
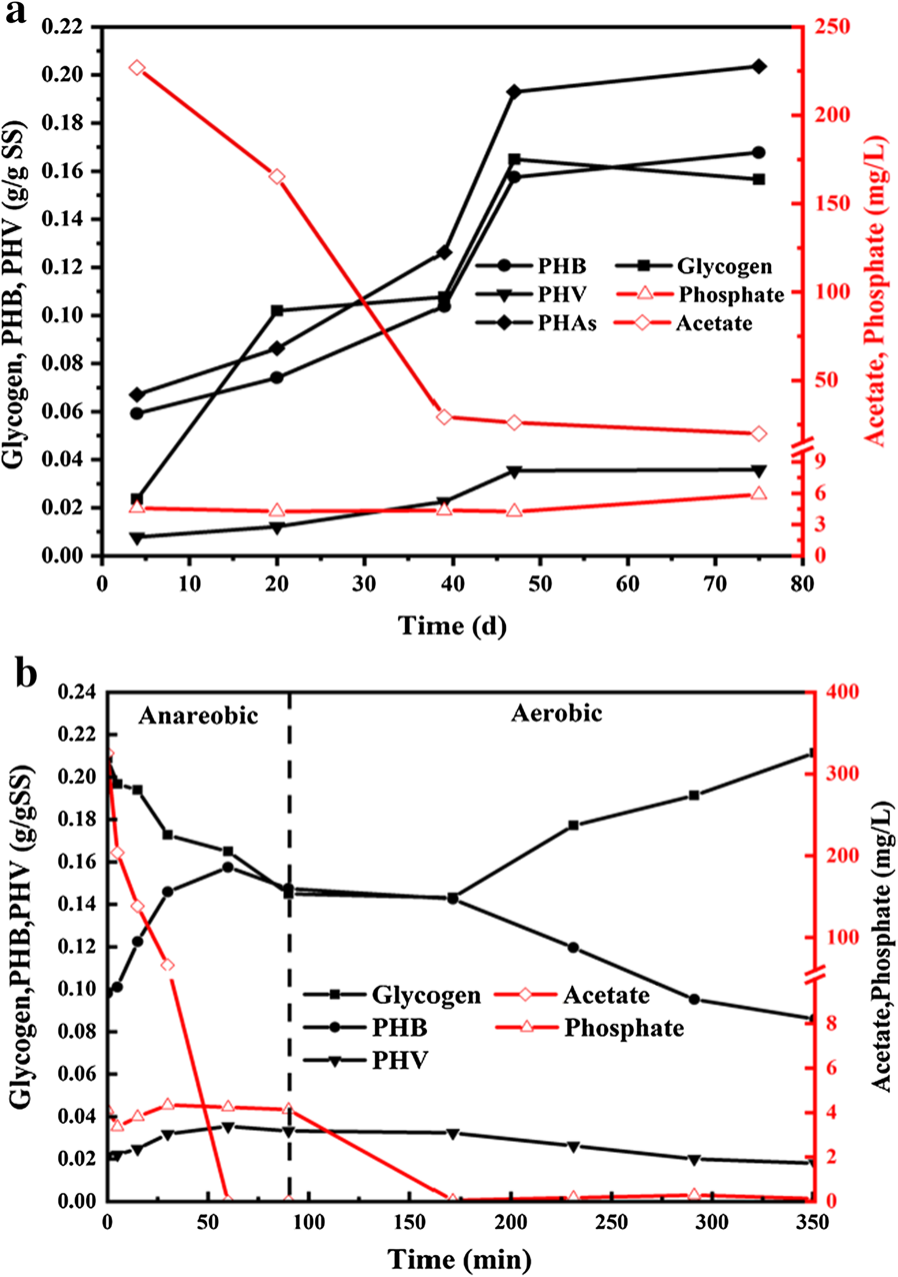
The performance of the GAOs enrichment culture system. **a** The changing levels of Glycogen, Acetate, PHAs and Phosphorus transformation during the 75 days operation. **b** The change levels of Glycogen, Acetate, PHAs and Phosphorus transformation in a typical cycle of the steady phage

The seeding sludge was collected from a secondary sedimentation tank of Tianjin wastewater treatment plant. In the process of sludge domestication, some indexes including acetate, glycogen, PHAs (PHB and PHV) and phosphate were performed. The long-term operation showed that the level of glycogen and PHAs (PHB and PHV) increased with the increasing time at the point of A60 (60 min of anaerobic), while the level of acetate decreased with the time. The level of glycogen synthesis also increased. Furthermore, the level of phosphorus almost retained unchanged. These results suggested that the rate of acetate removal and the level of glycogen and PHAs synthesis gradually increased in the system during the anaerobic period. The system showed the characteristic of GAOs after 39 d (Fig. 2a). The above indexes were also conducted in a typical anaerobic–aerobic cycle of the steady stage, the system showed the typical phenotype of a GAOs enriched culture during the anaerobic–aerobic phase. The results showed that acetate was completely consumed in 60 min of the anaerobic stage, which was accompanied by a decrease in intracellular glycogen levels by 0.064 g/gSS. PHB and PHV levels increased by 0.059 g/gSS and 0.012 g/gSS, respectively, while phosphate level remained almost unchanged, indicating that organic substrates were completely consumed and transformed into intracellular storage polymers PHAs (PHB, PHV). The level of phosphate retained at 5 mg/L from the beginning of anaerobic phase to the end of anaerobic phase, and there was almost no phosphate release during the anaerobic phase, implying that the energy enquired for the anaerobic uptake of acetic acid was derived from glycogen degradation, not from polyphosphate hydrolysis. In the subsequent aerobic phase, glycogen is accumulated and the intracellular polymer PHAs (PHB and PHV) are used for growth and to replenish glycogen stores (Fig. 2b). These results demonstrated that the typical characterization of GAM was successfully constituted.

In this study, we further compared the ratios of PHB/PHV production, glycogen consumption, phosphorous release, and acetic acid uptake to them of GAO/PAO models reported in the previous studies. The results indicated that the stoichiometric ratios were close to those of GAO or PAO models with glycogen accumulating metabolism (GAM). The ratios of Gly/VFA and PHAs/VFA were slightly higher than those reported by other GAO models, while PHV/VFA and PHV/PHB ratios were lower, for example, the ratio of PHV/PHB was 0.22 (Table 1), suggesting that we successfully established the GAOs enrichment model.

**Table 1:** Comparison of GAO and PAO models on ratios of PHB/PHV production, glycogen consumption, phosphorous release and acetic acid uptake

References	Model	SRT	pH	$\frac{P}{VFA}$	$\frac{Gly}{VFA}$	$\frac{PHB}{VFA}$	$\frac{PHV}{VFA}$	$\frac{PHAs}{VFA}$	$\frac{PHV}{PHB}$
This study	GAO	10 days	7.1 ~ 8.9	0.00	1.32	1.72	0.37	2.09	0.22
Zeng et al. (2003)	GAO	6.6 days	7 ± 0.1	NA	1.20	1.39	0.52	1.91	0.38
Lopez-Vazquez et al. (2007)	GAO	10 days	7 ± 0.1	0.01	1.20	1.28	0.69	1.97	0.54
Lu et al. (2006)	PAO	8 days	7.0 ~ 8.0	0.62	0.46	1.18	0.07	1.25	0.06
Acevedo et al. (2012)	PAO	8 days	7.0 ~ 8.9	0.73	0.35	1.30	0.06	1.36	0.05
Welles et al. (2016b)	PAOII-GAO-GAM	8 days	7.0 ± 0.1	0.03	1.28	1.45	0.5	1.95	0.34
Acevedo et al. (2012)	PAO-GAM	8 days	7.0 ~ 8.9	0.08	1.08	1.74	0.28	2.02	0.16
Acevedo et al. (2017)	PAO-GAM	8 days	7.0 ~ 9.0	0.05	1.19	1.31	0.63	1.94	0.48

### Microbial community dynamics in the GAO enrichment system

**Fig. 3:**
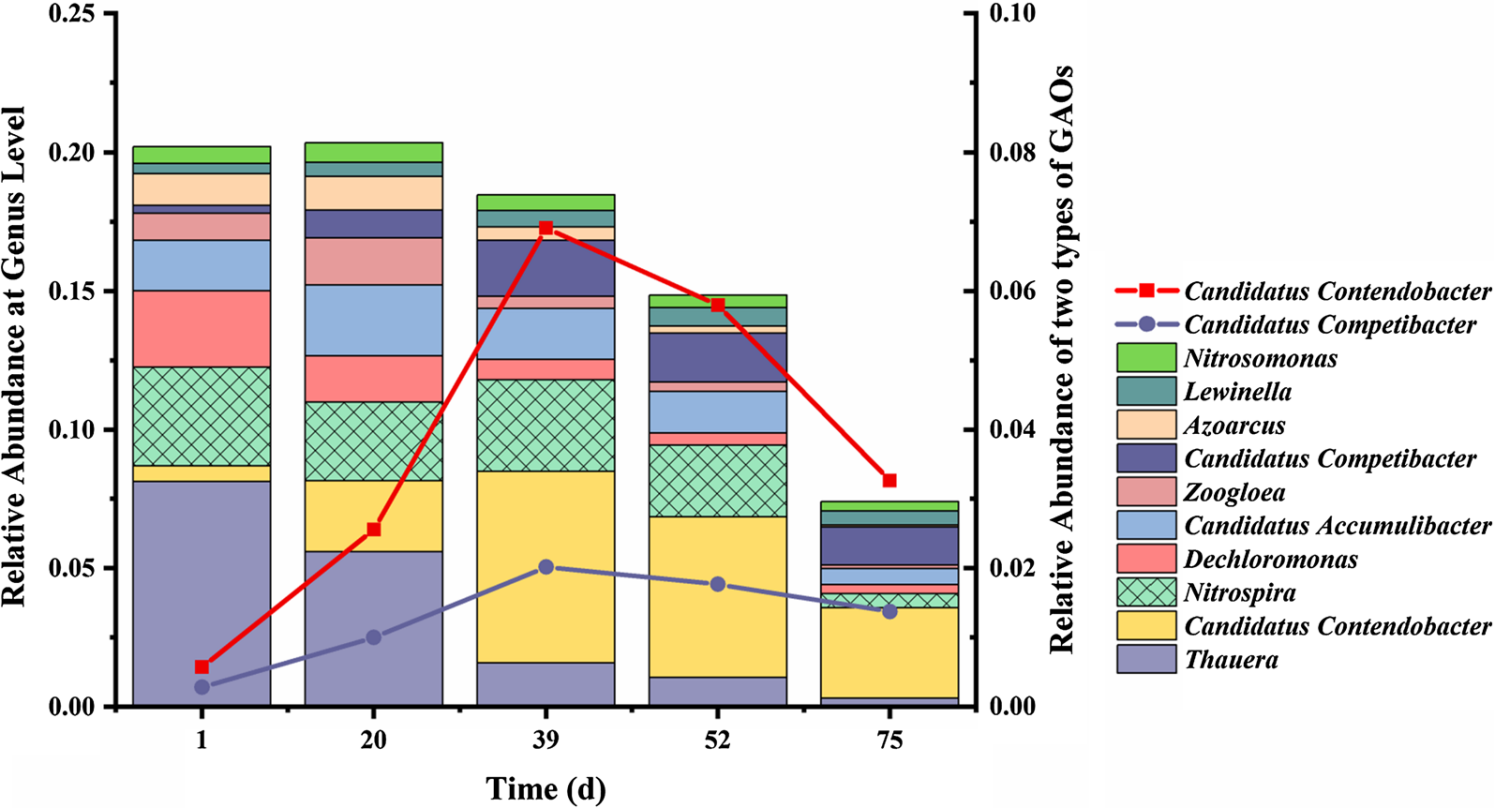
The relative abundance of the microbial community and the dominant GAOs at genus level in the process of reactor operation

To explore the distribution of microbial community and the dominant GAOs in the system, metagenomic sequencing was performed to quantify the microbial relative abundance at the genus level during reactor operation. The results showed that two kinds of GAOs *Candidatus Contendobacter* and *Candidatus Competibacter* were enriched, the relative abundance of *Candidatus Contendobacter* was higher from the beginning of the reactor operation to the stable stage of reactor than that of *Competibacter*, suggesting that *Candidatus Contendobacter* was the dominant GAO in the system (Fig. 3). At the beginning of reactor operation, *Thauera* was the dominant genus in the seeding sludge, followed by *Nitrospira*, *Dechloromonas*, *Candidatus Accumulibacter*, and *Candidatus Contendobacter*. From day 20 to day 39 of reactor operation, the relative abundance of *Candidatus Contendobacter* increased from 2.6 to 6.9%, and that of *Candidatus Competibacter* increased from 1.0 to 2.0%. On day 39, *Candidatus Contendobacter* replaced *Thauera* to become the dominant organism, and the relative abundance of *Candidatus Contendobacter* was 3.5 times higher than that of *Candidatus Competibacter*. Although the relative abundance of *Candidatus Contendobacter* slightly decreased after day 39, it continued to be the dominant organism in our system. These results further indicated that the characteristic of GAM contributed to the core community *Candidatus Contendobacter* in the system.

### The first five genera of the succinate-propionate pathway in the system

**Fig. 4:**
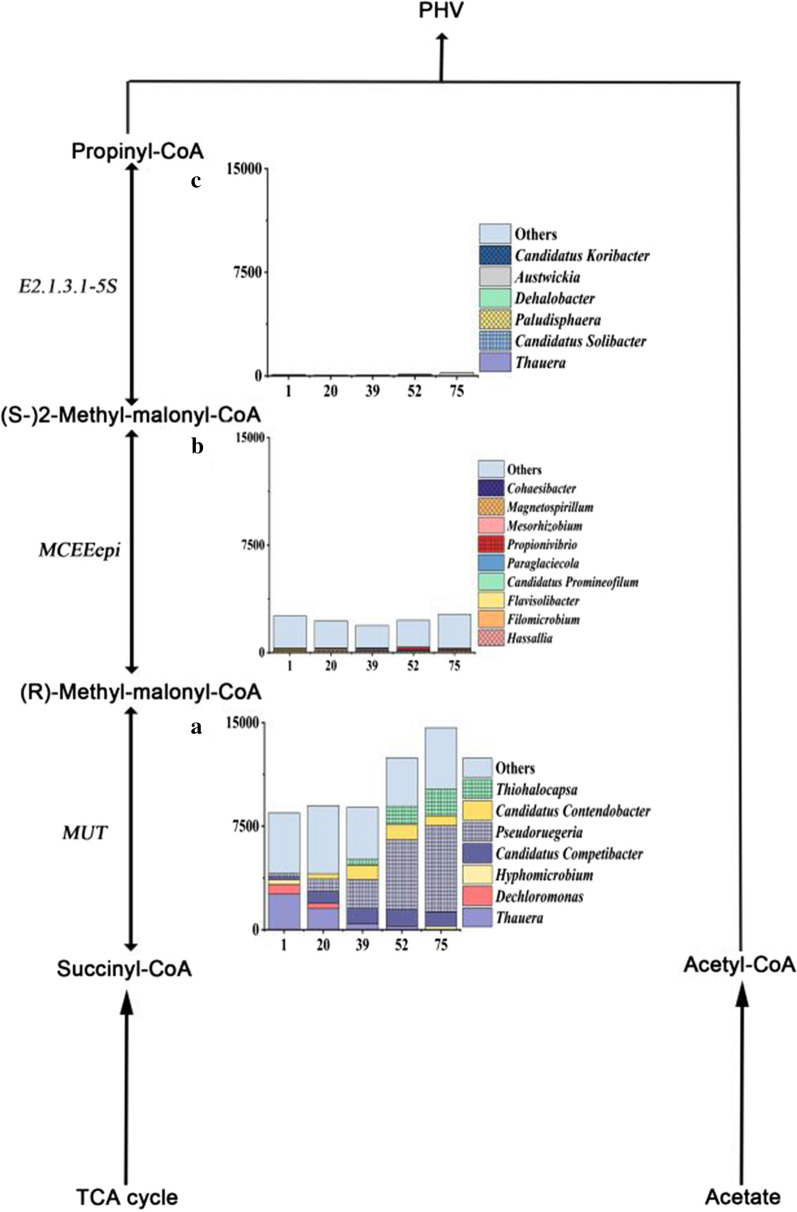
The changes of the abundance of the first five genera containing key genes that regulate propionyl-CoA production via the succinate-propionate pathway. **a–c** Represent the abundance changes of the first five genera for MUT, MCEEepi, and E2.1.3.1-5S, respectively

Previous studies had reported that the ratio of PHV/PHB was between 0.27 and 0.54, while the ratio of PHV/PHB was 0.22 in our system and far lower than that of the previous studies (Table 1), which suggested that the PHV synthesis pathway was different from previous studies (Filipe et al. 2001; Lopez-Vazquez et al. 2007). Therefore, it was essential to identify the relevant reactions of PHV synthesis and enable a validated model simulation. Propionyl-CoA was the key substance in the process of PHAs synthesis. To elucidate the origin of propionyl-CoA during PHV synthesis, we analyzed the source of key genes in the succinate-propionate pathway closely related to PHV synthesis. The succinate-propionate pathway converted succinyl-CoA produced by the TCA cycle to propionyl-CoA through three steps. The first step was that succinyl-CoA converted to (R)-methyl-malonyl-CoA, which was catalyzed by methylmalonyl-CoA mutase (*MUT*). In the succinate-propionate pathway of this study, *Candidatus Contendobacter* and *Candidatus Competibacter* were presented only in the first five (TOP5) genera of *MUT* gene. *MUT* gene showed a high relative abundance in *Candidatus Contendobacter* (0.0–11.4%) and *Candidatus Competibacter* (2.9–12.8%) (Fig. 4a) the results showed that *Candidatus Contendobacter* and *Candidatus Competibacter* could convert succinyl-CoA to (R)-methyl-malonyl-CoA. But the two key genes in other two steps, *MCEEepi* and *E2.1.3.1-5s*, were very low in the two types of GAOs (Fig. 4b, c). The results indicated that the abundance of key genes in the succinate-propionate pathway were low and changed slightly in the first five (TOP5) genera, suggesting the succinate-propionate pathway was not the advantageous pathway involved in PHV synthesis of *Candidatus Contendobacter* during the operating process.

### The first five genera of the EMC pathway in the system

**Fig. 5:**
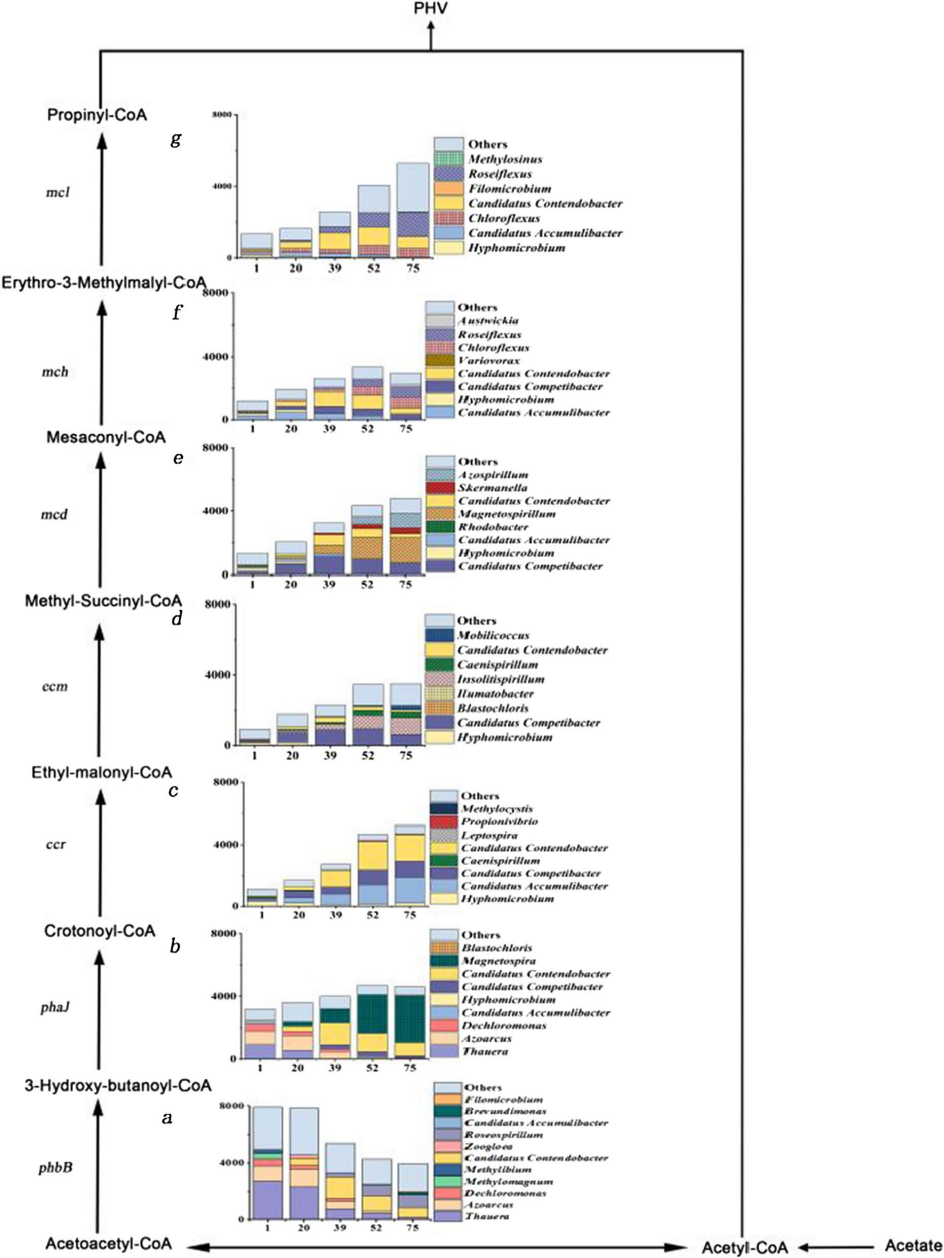
The changes of the abundance of the first five genera which containing key genes that regulate propionyl-CoA production via the EMC pathway. **a**–**g** Represent the abundance changes of the first five genera for phbB, phaJ, ccr, ecm, mcd, mch, and mcl, respectively

The EMC pathway has been reported as important sources of multiple coenzyme A including propionyl-CoA in *Methylobacterium extorquens* (Schneider et al. 2012), but the role of the EMC pathway in GAOs remained still unclear. In the EMC pathway, seven key genes including *phbB*, *phaJ*, *ccr*, *ecm*, *mcd*, *mch, mcl*, were involved in the synthesis of propionyl-CoA from acetyl-CoA, the abundance of top five (TOP 5) genera which containing the seven key genes changed markedly in the process of system operation. Metagenome analysis showed that the relative abundance of *Candidatus Contendobacter* ranged from 3.2 to 39.5%, which was significantly higher than that of other genera from day 20, and it was gradually increased with time. The relative abundance changes of *Candidatus Contendobacter* in the system were consistent with its relative abundance changes of the seven key genes including *phbB, phaJ, ccr, ecm, mcd, mch, mcl* in the EMC pathway (Fig. 5a–g). But *Candidatus Competibacter*, another GAO, contained only five regulatory genes including *phaJ, ccr, ecm, mcd, mch* in the EMC pathway (Fig. 5b–f), further indicating that *Candidatus Contendobacter* could synthesize propionyl-CoA independently through the EMC pathway.

**Fig. 6:**
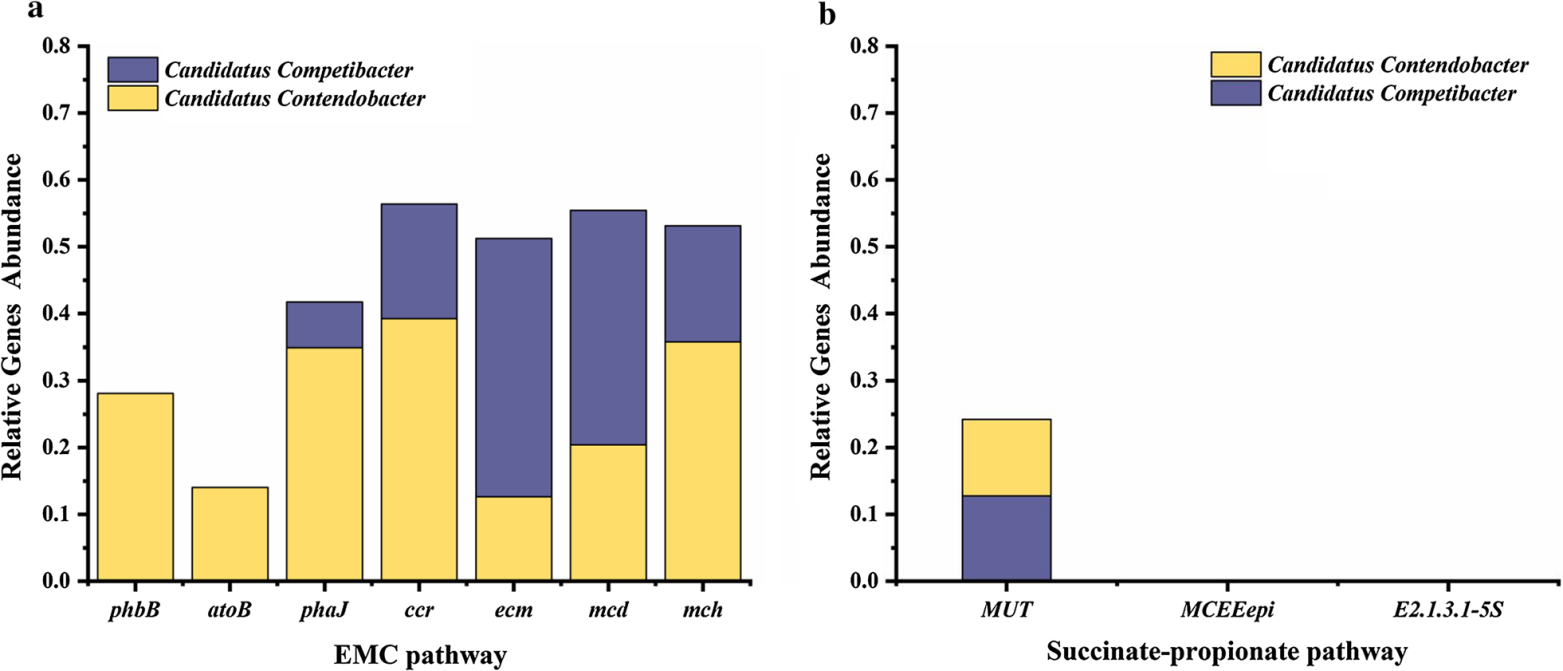
The analysis of key genes in *Candidatus Contendobacter* and *Candidatus Competibacter* on day 39. **a** Relative abundance of key genes of the EMC pathway. **b** Relative abundance of key genes of succinate-propionate pathway

On day 39, the results showed that the relative abundance of key genes was involved in the EMC pathways and succinate-propionate pathways in *Candidatus Contendobacter* and *Candidatus Competibacter* (Fig. 6a). *Candidatus Contendobacter* contained all seven key genes of the EMC pathway, whereas *Candidatus Competibacter* contained only five key genes of the EMC pathway. In contrast, *Candidatus Contendobacter* and *Candidatus Competibacter* only contained the *MUT* which was the first key gene of succinate-propionate pathways, while the other two regulatory genes, *MCEEepi* and *E2.1.3.1-5S*, were not present in the two types of GAOs (Fig. 6b), suggesting that EMC pathway played a crucial role in synthesizing PHV in *Candidatus Contendobacter*.

### The gene expression validation of propionyl-CoA synthesis pathway in *Candidatus Contendobacter*

**Fig. 7:**
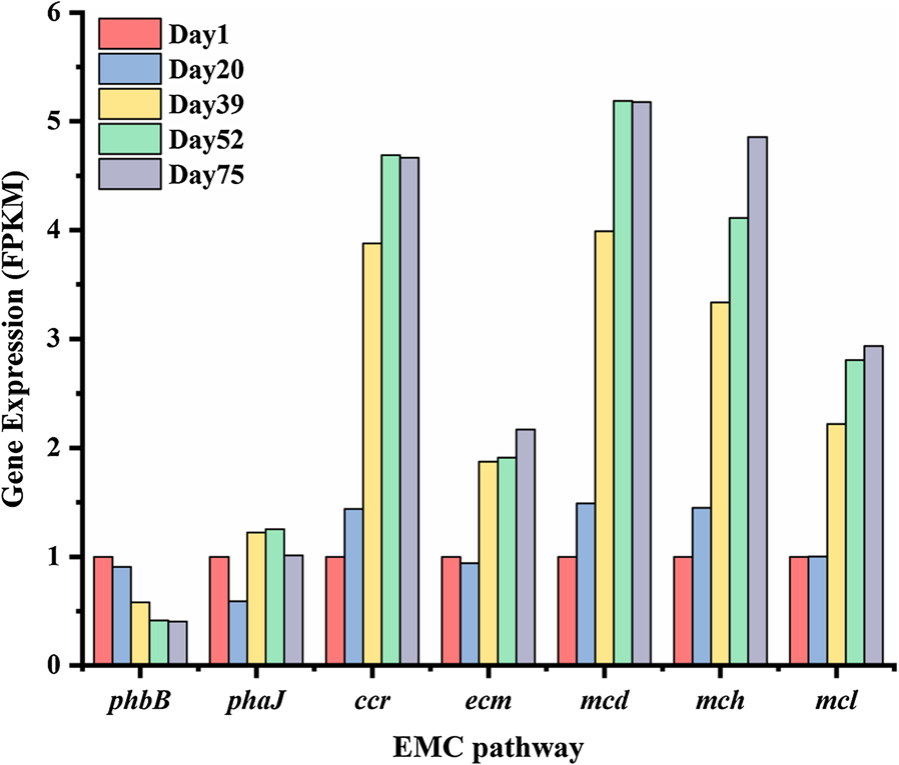
The changes of the key gene expression in the EMC pathway during the reactor operation

Although many studies had assessed PHV synthesis from metagenomic, few studies have analyzed it at the real-time expression level. To further validate the propionyl-CoA synthesis pathway in *Candidatus Contendobacter*, we conducted the expression levels of key genes in the EMC pathway at the transcriptional level after the system stabilization. Except for *phbB*, *phaJ*, the transcriptional activities of the remaining five genes, *ccr, ecm, mcd, mch, mcl*, were upregulated during reactor operation. The transcriptional expression levels of the two genes, *ecm* and *mcl*, were up-regulated by more than two-fold; the transcriptional expression levels of the three genes, *ccr, mcd,* and *mch,* were up-regulated by more than four-fold. The results indicated that the EMC pathway was generally more active as the abundance of *Candidatus Contendobacter* increased. The transcriptional expression of *ccr, mcd,* and *mch* genes was much higher than that of *phbB, phaJ, ecm, mcl* from day 39, indicating that these three genes are more actively expressed in the EMC pathway (Fig. 7). The results further validated that propionyl-CoA was synthesized through EMC pathway by *Candidatus Contendobacter*.

## Discussion

### The GAO enrichment model indicated the typical characteristic of GAM

In the previous EBPR systems, the presence of GAOs is of interest because their metabolism closely resembles that of PAOs, the only difference being the absence of phosphorus cycling (Roy et al. 2021). However, it has been challenging to identify PAO and GAO metabolisms and their role as GAOs in full-scale EBPR plants. In this study, *Candidatus Contendobacter* was the dominant GAO in our system rather than *Candidatus Competibacter* universally reported by many studies (Lanham et al. 2013; Ong et al. 2014). *Candidatus Competibacter* was the most common type of GAOs in both lab-scale reactors and full-scale wastewater biological treatment facilities (Nielsen et al. 2019; Yuan et al. 2020). *Candidatus Contendobacter* and *Candidatus Competibacter* were affiliated with *Competibacter*-lineage subgroups 5 and 1, respectively, suggesting that they might have different characteristics. In our system, *Candidatus Contendobacter* was the most dominant GAO, which could be because the operation mode was suitable for the growth of *Candidatus Contendobacter* rather than the growth of *Candidatus Competibacter*. Based on these findings, it could be concluded that the mode with decanting after the anaerobic period could effectively limit the growth of PAOs and successfully enriched the dominant GAO *Candidatus Contendobacter*.

Compared with the previous GAO models, there were many differences in our system, our model degraded more glycogen and synthesized more PHAs, and relatively less PHV. A reducing equivalent source, such as reduced nicotinamide adenine dinucleotide (NADH), was required under anaerobic conditions for the synthesis of PHA. Many studies (McIlroy et al. 2014) suggested that the excess reducing equivalents would be produced, when the amount of glycogen degraded anaerobically was greater than that of reducing equivalents required for the anaerobic uptake of acetic acid by GAOs. This excess was theoretically balanced with the flux of pyruvate through the reductive branch of the tricarboxylic acid cycle (TCA) and the succinate-propionate pathway to form propionyl-CoA. This ester was in turn condensed with acetyl-CoA to form PHV and was evidenced by the production of PHV from acetic acid in GAO enrichments (Oehmen et al. 2007). Based on the previous studies, compared with phosphorus accumulating metabolism (PAM), more PHV was produced via GAM, suggesting that PHV was obtained due to the consumption of excess reducing equivalents to balance internal reducing power (NADH) (Acevedo et al. 2012). The system degraded more glycogen in the system, theoretically, the excess reducing equivalents should be consumed by succinate-propionate pathway to produce more PHV, but the amount of PHV synthesis was lower than that reported by the previous models. Therefore, we had a reason to infer that the GAOs enrichment system with the dominant GAO *Candidatus Contendobacter* had its characteristic on the utilization of the excess reducing power for propionyl-CoA forming and PHV synthesis.

### The succinate-propionate pathway cannot contribute to PHV synthesis

PHAs, a class of biodegradable polymers, were synthesized as energy storage molecules by many bacteria (Mannina et al. 2020; Pisco et al. 2009). GAOs utilized glycogen to obtain energy and reducing equivalents for VFA uptake and PHA storage. Under aerobic conditions, glycogen is regenerated from PHAs, which then supplied energy and reducing power for GAOs growth. PHAs were generally synthesized from acetyl-CoA and propionyl-CoA (Bengtsson 2009; Zhang et al. 2019), both of which were assumed to randomly condense to PHB, PHV, and PH2MV (Filipe et al. 2001). One molecule of acetyl-CoA combined with one molecule of propionyl-CoA to form PHV monomer, which in turn condensed to form PHV (Guedes da Silva et al. 2020).

Propionyl-CoA can be synthesized via two potential pathways: succinate-propionate pathway and ethylmalonyl-CoA (EMC) pathways (De Meur et al. 2018; Schneider et al. 2012). In the former, propionyl-CoA was synthesized via succinyl CoA and 2-methyl-malonyl-CoA, which were TCA cycle intermediate metabolites (McIlroy et al. 2014), and in the latter, it was synthesized upon the conversion of acetyl-CoA. Finally, PHV was synthesized via propionyl-CoA combined with acetyl-CoA. In this study, genes source analyzed the relation between the three key genes of the succinate-propionate pathway and the first seven community of the system. The results indicated that *MUT* gene could locate in *Candidatus Contendobacter* and *Candidatus Competibacter* and had a high relative abundance. But the other two regulatory genes, *MCEEepi* and *E2.1.3.1-5s*, were not present in the two types of GAOs and had a significantly low relative abundance, especially *E2.1.3.1-5s* gene (Fig. 6b). These results implied that it was challenging for GAOs to synthesize propionyl-CoA via the succinate-propionate pathway in our system.

### The EMC pathway: a novel propionyl-CoA synthesis pathway for PHV in *Candidatus Contendobacter*

In the present study, genes source analysis indicated that the seven key genes of the EMC pathway could completely located at *Candidatus Contendobacter*, whereas *Candidatus Competibacter* contained the only five key genes of the EMC pathway (Fig. 6a), suggesting that propionyl-CoA in *Candidatus Contendobacter* could be synthesized through the complete EMC pathway. *Candidatus Contendobacter* predominantly produced propionyl-CoA through the EMC pathway, which in turn supplied the substrates for PHV synthesis. The relative abundance of different key genes including *phbB*, *phaJ*, *ccr*, *ecm*, *mcd*, *mch*, *mcl* in the EMC pathway considerably varied in *Candidatus Contendobacter*. The previous studies had reported that the seven genes were located at the key nodes in the EMC pathway and played an important role in regulating the core enzymes of the EMC pathway (De Meur et al. 2018; Schneider et al. 2012). But *Candidatus Competibacter* expressed the only five core genes in the EMC pathway, it could not synthesize propionyl-CoA or PHV independently, which further suggested that *Candidatus Contendobacter* was mainly responsible for PHV production through the EMC pathway in our system.

Compared to the classic GAO model (Table 1), we also found that glycogen degradation was greater in the anaerobic phage than that in other GAO models, one reason could be that the EMC pathway in *Candidatus Contendobacter* produced lower levels of propionyl-CoA to balance an equivalent amount of reducing power than the succinate-propionate pathway; another reason could be that the EMC pathway had more intermediates participated in other pathways. For example, mesaconyl-CoA in the EMC pathway also participated in the glyoxylate and dicarboxylate metabolism pathway, which could affect the amount of propionyl-CoA synthesis in different GAO models. *Candidatus Contendobacter* in our system was more inclined to invoke the EMC pathway to synthesize propionyl-CoA, consequently playing a pivotal role in PHV synthesis. Genes source analysis also showed that *Candidatus Contendobacter* was the main bacterium that invoked genes expression of EMC pathway in the system. In this manner, *Candidatus Contendobacter* synthesized the key substrate for PHV synthesis (propionyl-CoA) through the EMC pathway.

In summary, we successfully constructed GAOs enrichment system, which showed the classic GAM from the dominant organisms *Candidatus Contendobacter*. We found that the ethylmalonyl-CoA (EMC) pathway was the crucial pathway supplying propionyl-CoA for poly-β-hydroxyvalerate (PHV) synthesis in *Candidatus Contendobacter*. Gene source analysis showed that the genes of EMC pathway expression increased with *Candidatus Contendobacter* enrichment, further validating that propionyl-CoA was synthesized by *Candidatus Contendobacter* predominantly via EMC pathway. Our work revealed the novel mechanisms underlying PHV synthesis through EMC pathway and improved the intercellular storage metabolism of GAOs.

## Data Availability

The datasets generated during and analyzed during the current study are available from the corresponding author on reasonable request.
